# A Brief Report on Reviews of Existing Creative Art–Based Interventions in Dementia Care From 2010–2020

**DOI:** 10.3389/fragi.2022.865533

**Published:** 2022-04-28

**Authors:** Irfan Manji, Pascal Fallavollita

**Affiliations:** ^1^ Interdisciplinary School of Health Sciences, Faculty of Health Sciences, University of Ottawa, Ottawa, ON, Canada; ^2^ School of Electrical Engineering and Computer Science, Faculty of Engineering, University of Ottawa, Ottawa, ON, Canada

**Keywords:** creative arts, dementia care, Alzheimer’s disease, reviews, brief report

## Abstract

The following brief report provides an overview of previously published reviews in the context of creative arts-based interventions for persons with dementia. A total of 22 review articles were identified and summarized. Next steps are suggested for future studies that may wish to a) develop a new review, or b) create new studies filling in the gaps identified by the authors in this report.

## 1 Introduction

### 1.1 Dementia

Dementia impacts over fifty million people worldwide (World Health Organization, 2020). With growing numbers each year, alternative modes of interventions are constantly needed to counteract or aid in alleviating health issues associated with dementia. Adult brain functions, including memory, judgment, reasoning, behavior, and emotions (Alzheimer Society, 2016), have all been noted to impact the person.

### 1.2 Creative Art–Based Interventions

Care providers postulate that pharmacological treatments should be avoided and only used sparingly in dementia care (Douglas et al., 2004 as cited in Guseva, 2019), Therefore, for creative arts, nonpharmacological interventions should be used as they provide various benefits such as symptom, behavior, or cognitive function management (Cerejeira et al., 2012; Guseva, 2019), improve well-being and/or quality of life (Cohen-Mansfield et al., 2011; van Dijk et al., 2012; Burnside et al., 2017), and target improvement in overall health for persons with dementia (Zeilig et al., 2014).

The creative arts can foster creativity, agency, and increase autonomy and engagement in persons with dementia (Chancellor et al., 2014; Meekums et al., 2015). Creative arts are seen in the form of art, music, dance, and theater/drama interventions (Demarin et al., 2016; Megranahan and Lynskey, 2018; Social Care Institute for Excellence, 2020). Recently, various researchers have referred to creative art–based interventions as psychosocial (Lawrence et al., 2012; Guseva, 2019) or sensory-based (Smith and D’Amico, 2020) interventions.

Opportunities for creativity and creative expression remain intact even as dementia progresses (Crutch et al., 2001; Miller and Miller, 2013). Creativity supports positive aging and provides structure and purpose to the person (Price and Tinker, 2014). Furthermore, the capacity to be creative and to provide the necessary environment to express creative endeavors reinforces that each person has access to artistic and creative abilities as it is developmentally encoded in each person, including those with dementia (Palmiero et al., 2012; Miller and Miller, 2013). The work of Maduro (1974) established that artistic creativity does not decline with chronological age as it can peak in early middle age and remain vibrant in later life. As a result, being creative means to participate (Maduro, 1974) and promotes positive aging (Price and Tinker, 2014).

The nuances of creativity are notably discussed by Glãveanu (2013), Kaufman and Beghetto (2009), and Kharkhurin (2014). These authors all evaluate and explain the creativity criteria that are present even as an individual ages, or ages with dementia. Glãveanu (2013) speaks of the 5° A’s (actors, actions, artifacts, audiences, and affordances); Kaufman and Beghetto (2009) have their 4 C model (little-c, big-c, mini-c, and pro-c); and Kharkhurin (2014) has four criteria for creativity (novelty, utility, aesthetics, and authenticity). In relation to creative art–based interventions, creativity provides a tangible expression of self and shows that even with dementia, there is a motivation to express artistic creativity (Crutch et al., 2001) that can reinforce a positive self-image (Ullán et al., 2012).

### 1.3 Reviews

Many authors have summarized and reviewed creative art interventions through scoping and systematic reviews. Reviews, in general, narratively or meta-analytically, synthesize and disseminate key findings that provide policy makers and researchers with data points to build more or better studies for the target population. Scoping reviews provide a broad picture of the body of literature and give an overview of emerging evidence which can inform practice in the research field (Munn et al., 2018). Systematic reviews aim to systematically summarize evidence relating to efficacy (Liberati et al., 2009) and minimize bias using methods that can be documented in advance with a protocol (Chandler et al., 2021).

### 1.4 Purpose

As the number of individuals diagnosed with dementia increases, there is an amplified strain on dementia care. Not only are pharmacological routes being used, but in recent times, more creative art–based interventions are being implemented and shared in settings with persons with dementia. It is important to continue providing robust research in this field of work as creative arts provide a positive impact on the target population. Furthermore, reviews provide a snapshot of the literature and ask important research questions that are designed to list gaps found in the current state of the literature. To our recollection, we have yet to see a brief report summarizing the last 10 years of review data. As a result, the motivation to undergo the work presented here was to look at which current reviews are related to the health and care of persons with dementia through the creative arts. We summarized reviews from 2010–2020 and provided further steps for future reviews or for future studies wishing to undergo creative art–based interventions for persons with dementia.

## 2 Materials and Methods

### 2.1 Eligibility

#### 2.1.1 Population

Our target population was persons with dementia (PwD) or Alzheimer’s disease. No limitations were placed on the population based on age, sex, gender, ethnic or racial background, type of dementia or stage of disease, or on type of review.

#### 2.1.2 Intervention

We examined creative art–based interventions from 2010 to 2020, which can be considered as art, music, dance, and theatre/drama interventions (Social Care Institute for Excellence, 2020).

#### 2.1.3 Outcome

Key findings and aims from the included reviews were extracted in order to suggest what elements future review or research works may consider incorporating.

#### 2.1.4 Other

Only articles in English were included after the title and abstract screening.

### 2.2 Search Strategy and Information Sources

The search strategy focused around three main search concepts: the intervention (creative arts), population (dementia/Alzheimer’s disease), and reviews. Furthermore, review articles from the past decade (2010–2020) were identified from our searches. The returned review articles were then screened at the title and abstract level by the first author (blinded) and were managed in Microsoft Excel.

A literature search using databases PubMed, Scopus, and Medline was conducted on 25 December 2020. A second literature search was conducted on 5 July 2021 to ensure that we had not missed any studies that had been published in the interim. Table 1 contains an example of the search strategy used in PubMed.

**TABLE 1:** Search strategy in PubMed for creative art therapy reviews.

Creative art–based intervention*	Population	Review type
Art*	Dementia OR Alzheimer’s*	Systematic OR scoping OR rapid OR realist

*Other therapy synonyms searched were: theatre or drama, music*, and dance*, as well as their synonyms. For example, in the case of dance, terms such as dance*, dancing*, and movement were also searched.

## 3 Results

### 3.1 Previously Published Reviews

After examining the title and abstracts from the searches in Table 1, a total of 22 articles that focused on the PwD and creative art–based interventions were included. A significant number of art reviews (41%) have been performed in the past 10 years. The art reviews focused on visual arts (Chancellor et al., 2014), participatory arts (Zeilig et al., 2014; Ward et al., 2020; Cavalcanti Barroso et al., 2022), classified creative arts under the umbrella term of art therapy (Beard, 2011), or nonpharmacological interventions (Brown Wilson et al., 2019). The remaining creative arts were represented as music (27%), dance (23%), psychosocial (4%), and sensory (5%) interventions. No review articles outright developed a theatre/drama-related review or museum-led review.

All included studies were summarized, and data were extracted from the articles to ascertain the gaps to provide the next steps. Supplementary File SA contains essential data points from each review article. Data extraction items such as study details, setting, population, research aims, eligibility criteria in the reviews, number of included studies, and key findings are included in this file.

### 3.2 Characteristics of Included Studies

The main characteristics of creative art–based interventions found in the included review articles for PwD are as follows:

• The authors looked at how the creative arts influenced decreases or increases in the symptoms of dementia; in other words, symptomatic changes (Cowl and Gaugler, 2014; Aleixo et al., 2017; Karkou and Meekums, 2017; van der Steen et al., 2018; Brown Wilson et al., 2019; Clare and Camic, 2019; Dowson et al., 2019);
• Most search strategies were isolated for randomized controlled trials (Blackburn and Bradshaw, 2014; Karkou and Meekums, 2017; Deshmukh et al., 2018; van der Steen et al., 2018; Brown Wilson et al., 2019);
• The review articles focused on multiple settings (e.g., community and nursing homes) (Chatterton et al., 2010; Aleixo et al., 2017; Deshmukh et al., 2018; van der Steen et al., 2018; Clare and Camic, 2019);
• Some articles did not clearly state the setting they were investigating (Beard, 2011; Chancellor, et al., 2014; Cowl and Gaugler, 2014; Dowson et al., 2019; Jiménez et al., 2019; Klimova et al., 2017; Mabire et al., 2019; Ruiz-Muelle and López-Rodríguez, 2019; Salisbury et al., 2011; Zeilig et al., 2014);
• Finally, two articles placed more emphasis toward the caregivers, staff, or therapists (Chatterton et al., 2010; Smith and D’Amico, 2020).

Some reviews returned low included study results, which could be explained by either a narrow search strategy/eligibility criteria or due to the paucity of available research at the time of their review. Some reviews suggested that creative arts interventions are positive interventions that can help people with dementia in various facets of their life. However, the low amount of health or well-being oriented studies (Zeilig et al., 2014; Dowson et al., 2019; Ward et al., 2020) shows that even though we are moving toward a holistic approach with the creative arts, the state of the literature remains steadfast on the various symptoms and behavioral disturbances that are associated with dementia instead of focusing on the needs of the person.

## 4 Discussion

### 4.1 Next Steps: New Review Works

Supplementary File SC identifies potential further steps, which were based on gaps found within the review articles. These next steps may be undertaken to help generate new search strategies prior to developing reviews using creative art–based interventions for PwD.

A few suggestions will be summarized here. Beard (2011) had isolated the literature from 1990 to 2010; therefore, a gap exists in the last 10 years of data on creative art–based interventions. Furthermore, some reviews (Beard, 2011; Ruiz-Muelle and López-Rodríguez, 2019) chose to include only persons with Alzheimer’s disease. This form of dementia makes up 60%–70% of the cases worldwide (World Health Organization, 2020) but still leaves 30%–40% accounting for other forms. A future search strategy should capture key words such as dementia and Alzheimer’s disease to be more inclusive of the entire population.

The findings in the works by Aleixo et al. (2017) and Dowson et al. (2019) mainly evaluated music therapy on neuropsychiatric symptoms. However, Dowson et al. (2019) suggested that music can also support and strengthen relationships, provide in-the-moment experiences, and meet the psychosocial needs of people with dementia. The next steps would be to create a design that is not focused only on symptomatic improvement but rather focuses on the person, as seen in the residual impacts from Dowson et al. (2019). Further studies could also benefit from investigating the principles of personhood (Kitwood, 1997a, b), selfhood (Sabat, 2002, 2005), or embodied selfhood (Kontos, 2004, 2005), which may complement research in understanding the person and their needs more than the ailments associated with the condition.

A total of six out of the 22 reviews focused on research containing randomized controlled trials (RCTs). Out of the six, three reviews yielded less than seven studies per review (*n* = 0, *n* = 2, *n* = 6). One of the reviews that investigated RCTs (Karkou and Meekums, 2017) found zero studies. Their search strategy required that only RCTs be included, particularly those that assess the effects of dance movement therapy on symptoms of dementia compared to no treatment, standard care, or other treatment (Karkou and Meekums, 2017). They also wanted to compare different forms of dance movement therapies (Karkou and Meekums, 2017). Yielding zero studies shows that the authors or another group of researchers can remove the RCT limit and direct the review to encompass multiple research designs or zone in on another design. A similar approach was undertaken in the works by Deshmukh et al. (2018) (*n* = 2) and Blackburn and Bradshaw (2014) (*n* = 6). See Supplementary File SA for more details on their inclusion and exclusion criteria. It is worth noting that RCTs are an effective methodology as they are considered the gold standard in research (Spieth et al., 2016; Hariton and Locascio, 2018). They require careful and rigorous planning and coordination (Bondemark and Ruf, 2015). RCTs have shed light on many aspects of psychosocial interventions for people with dementia, as seen from the studies of the included reviews in this report. However, there are instances when researchers may not be able to conduct an RCT. For example, Sauer et al. (2016) were unable to conduct an RCT as the long-term care facility did not allow for residents to be excluded. As a pivot, Sauer et al. (2016) created a subsample of their original population from those who were available.

Future reviews could consider including studies that use subsample populations where RCTs are not possible. In this way, researchers must comprehensively decide which study design works for them. The same level of thinking goes into the development of reviews. Furthermore, researchers aiming to understand the lived experiences of a traumatic event from six individuals versus comparing multiple interventions on groups of individuals require thorough thinking and implementation of the correct research methodology. For instance, qualitative methods may be more appropriate for the type of study being conducted.

There was a wide range of assessment tools used in the studies found in the reviews. The tools that assessed the symptoms of dementia or mental state were the Mini-Mental State Examination (*n* = 11), Cohen-Mansfield Agitation Inventory (*n* = 7), Geriatric Depression Score (*n* = 7), and the Neuropsychiatric Inventory (*n* = 9). The most common tools that focused on the interaction with the creative art–based interventions were the Greater Cincinnati Chapter Well-Being Observation Tool (*n* = 3) and Dementia Care Mapping (*n* = 3).

One author concluded that the range of assessment tools available, for the most part, were problematic in nursing home settings due to individuals having multiple comorbidities associated with medical and psychiatric health (Brown Wilson et al., 2019). Brown Wilson et al. (2019) also stated that the measurement tools were not often designed to test such populations (those with many symptoms). Another said that these tools were used inconsistently across their pool of included studies (Beard, 2011). These conclusions are concerning as we noticed that majority of the instruments were not measuring the interactions between the PwD and the creative art–based intervention they were participating in; there was focus on decreasing the many symptoms of dementia, which are equally as important. However, we wonder if the current state of outcomes from these reviews is heavily saturated as majority of the outcomes are associated with symptomatic change instead of visualizing quality of life and well-being to include more than the symptoms of the individual. Refer to the works of Kitwood, Sabat, and Kontos. A full list of assessment tools found by the review studies is located in Supplementary File SB *.*

Future studies looking to develop assessment tools in their work may turn their attention to the rigorous work provided by Algar et al. (2016), Bowling et al. (2015), Missotten et al. (2016), and Ready and Ott (2003). These authors have summarized various assessment tools and rationalize that the tools are chosen based on the definitions and aims of the study.

Finally, some published reviews required that a creative art–based intervention be administered and monitored by a professional therapist (Chatterton et al., 2010; Karkou and Meekums, 2017; Brown Wilson et al., 2019). This approach may exclude primary caregivers or staff members at local residential facilities and could also be costly. In addition, comfort levels may vary among PwD; therefore, not all interactions could be valued as being pleasant. The next step would be to expand the type of intervention provider to not only trained therapists but also to caregivers, staff members, and any other individual that may be around the PwD. This may increase comfort and provide positive reactions to the interventions.

There were no theatre/drama-based reviews found for this brief report. Common theatre/drama interventions have been seen in medical clowns (Raviv, 2014; Ramgard et al., 2016), elder clowns (Warren and Spitzer, 2011; Kontos et al., 2017), or in ‘play’ (Swinnen and de Medeiros, 2018). The data and studies are there, but no theatre/drama-based endeavors reviews were found. One may consider undergoing a scoping or systematic review to evaluate the work being done currently on theatre/drama. Furthermore, no included reviews for this brief report solely focused on museum interventions. The Social Care Institute for Excellence (2020) acknowledges that museums engage with PwD and can improve well-being though stimulation of memory and conversations. Museums also provide space for reflection as PwD can explore feelings, memories, desires, and thoughts, without being restricted to a medical setting (Cunniffe, 2019).

### 4.2 Limitations

In our search strategy and subsequent title and abstract screening, only articles written in English were chosen. This is a limiting factor that potentially overlooks review articles from other languages. However, due the lack of a translator and funding costs, we were unable to include other language-based articles.

## 5 Conclusion

In summary, the current need is for new reviews to include all PwD (dementia and Alzheimer’s disease as key words in strategies), explore into the persons and their creativity more than the condition, expand on the type of study designs, incorporate a variety of creative art facilitators, and be inclusive of assessment tools which can track changes in the symptoms and the impact of the creative arts on the population.

Extensive bodies of work on reviews pertaining to creative art–based interventions have been published in the last 10 years; however, gaps still exist in the current state of review work as found through our suggestions for further steps. The next steps were suggested not only for new review works but also for future researchers contemplating future studies. We hope that they are able to take multiple directions with their research. This brief report illustrates how research and review work in dementia care is undeniably important and needed, especially through creative nonpharmacological interventions. Further works may benefit from designing reviews from some of the gaps identified in the next steps.

## Data Availability

The original contributions presented in the study are included in the article/Supplementary Material, further inquiries can be directed to the corresponding author.
