# Supplementary material for: A Brief Report on Reviews of Existing Creative Art–Based Interventions in Dementia Care From 2010–2020
Source: Front Aging. 2022 Apr 28;3:865533. doi: 10.3389/fragi.2022.865533 (PMC9261444; doi:10.3389/fragi.2022.865533)
Supplement: Supplementary file 1 [file Table1.pdf]

Supplementary File SA

Previous Review Works on Creatives Arts-based Interventions for People with Dementia summarized.

Art Therapy

Study	Setting	Population	Research Aims	Eligibility	Included Studies	Key Findings
Beard (2011)	Not stated	Alzheimer's disease	This review aimed to critique evidence base of art therapies including music, visual arts, drama, and dance/movement. The author asked four research questions:<br><br>1. What is the focus of the empirical base on using AT with persons who have DAT?<br>2. (How) have these studies been designed and evaluated?<br>3. What findings are reported?<br>4. What does this tell us about the potential of AT for enriching the lives of people with dementia?	Inclusion:<br>• evidence base of art therapies (music, visual arts, drama, dance/movement) from 1990 to 2010	Unclear	Empirical weakness:<br>unclear study designs, activity descriptions, methods used; poorly identified or unspecified; too much emphasis on clinical outcomes; a lack of systematic analysis of data.<br><br>Omissions within dementia-specific work: lack of accounts self- reports; lack of tailored therapies to persons in the early stages versus late-stage; imbalanced attention between persons living at home versus nursing facilities; product focused versus process focused or quality of life focused.
Brown Wilson et al. (2019)	Nursing Home	Dementia	This review aimed to assess the effectiveness of nonpharmacological interventions used in reducing anxiety symptoms. The authors asked:<br>1. Are nonpharmacological interventions effective in reducing anxiety symptoms in	Inclusion:<br>• older people with dementia, Parkinson's disease with dementia<br>• residential aged care, Assisted living, specialist dementia unit<br>• psychotherapeutic intervention<br>• randomized control trials<br>• use of a validated tool that measures anxiety<br>• English only<br><br>Exclusion:	13	Few studies looked at anxiety as the primary outcome; some studies considered anxiety as part of the construct for mood, or behaviour and psychological symptoms of dementia.<br>Anxiety was only a primary outcome in studies focused on a music intervention. Music therapy was the most consistent approach in reducing anxiety (six out of thirteen included articles).

			older adults with comorbid dementia?<br>2. If so, which nonpharmacological interventions demonstrate the greatest effectiveness in reducing anxiety symptoms in older adults with comorbid dementia?	• people without dementia, people with mild cognitive impairment,<br>• caregivers<br>• other mental health issues<br>• population-based studies<br>• home care, primary care, memory clinic, Inpatient or outpatient of a hospital, Community-dwelling<br>• drug intervention<br>• individual case studies/case reports<br>• prevalence studies<br>• case–control studies, Randomized control trial protocol, Narrative reviews or systematic reviews, Opinion pieces or commentary<br>• global or subjective assessment of anxiety<br>• cognitive assessment only		
Cavalcanti Barroso et al. (2020)	Not stated	Dementia	Report and summarize on the effects of visual participatory arts activities in dementia research. Evidence included quantitative, qualitative, and mixed-methods study designs.	Inclusion:<br>• Art should be created by the person and not just observed or discussed<br>• Empirical evidence using quantitative, qualitative, and mixed-methods designs<br>• People living with dementia and participatory art interventions, peer-reviewing (in English), and publications before Jan 2019<br><br>Exclusion:<br>• Research without participants with dementia or related cognitive disorders, other interventions not visual arts related<br>• cases studies/dissertations/conference papers	20	Participatory visual arts have a positive effect on cognition, social, and psychological functioning of people with dementia. Although, the heterogeneity of the studies provided an inconsistent evidence of an overall positive effect. Participants thought interventions were enjoyed and engaging.<br><br>Included studies were classified into the following outcome areas: program evaluation, psychological aspects, well-being, and social aspects.
Chancellor, et al. (2014)	Not stated	Alzheimer's Disease and other dementias	The authors explored art therapy's usefulness in dementia.	Inclusion:<br>• years 1980 to 2013<br>• visual art therapy	16	Proposed a three-level framework on art therapy for persons with dementia: art therapy relies on preserved abilities and not on correcting disabilities; allows for emotional expression, especially when verbal communication is a difficulty; visual art opens a state of 'flow', potentially enhancing well-being.

Cowl & Gaugler (2014)	Not stated	Alzheimer's Disease and Dementia	The goal of this review is to determine the efficacy and benefits of arts therapies (visual arts, music, drama, dance movement, songwriting, and poetry) for persons with Alzheimer's disease and dementia.	Inclusion:<br>• in English<br>• creative arts therapy as at least one of the interventions<br>• participants w AD/D<br><br>Exclusion:<br>• unspecified participants for whom outcomes were measured or developed<br>• not specified with Alzheimer's disease or dementia<br>• not original work<br>• described changes or characteristics of individuals' artwork as their disease progressed without describing the therapeutic benefits of art participation for the individual	112	Data was split into quantitative and qualitative works.<br><br>Twelve out of fourteen randomized control trials provided that creative art therapies are potentially effective.<br><br>The three categories of outcomes (cognitive, behavioural, and emotional) varied in capacity to improve symptoms in participants.
Deshmukh, et al. (2018)	Community or any form of institutional care	Dementia	Aimed to review the effects of art therapy as an adjunctive treatment for dementia compared to standard care and other non-pharmacological interventions.	Inclusion:<br>• randomized controlled trials<br>• participants with a formal diagnosis of dementia<br>• intervention was to be a minimum of five sessions	2	Limited information on the effectiveness of art therapy due to the small number of studies included.
Salisbury et al. (2011)	Not stated	Dementia	Scoping review aims to identify published and grey literature relating to art therapy program effectiveness, review and summarize research evidence, and identify gaps in the research.	Inclusion:<br>• peer reviewed<br>• in English<br>• available in March 2010<br>• grey reports	42	Themes seen: promoting well-being, reducing isolation, positive effects and cost savings, arts appreciation, self-expression, Art therapy, Rewarding the brain, and Preventing cognitive decline<br><br>Benefits seen were: an increase in quality of life and well-being, improved communication (non-verbal and verbal), reminiscence opportunities, and encouraging meaningful conversation, sense of self was regained, and helped carers gain an insight into the experiences of those they care for.
Ward et al. (2020)	Community	People with dementia	This review had the following objectives:<br>• offer a descriptive overview of the different participatory arts<br>• consider holistic benefits of participatory arts by	Inclusion:<br>• people living with early to moderate stages of dementia in the community<br>• carers or partners of people living with early or mid-stage dementia.<br>• participatory arts-based interventions (singing, playing, and making music, dancing and movement, drama and	26	Three key areas of interest: *The Individual; The Group*; and *Space, Place and Objects*.<br><br>The individual focused on: person-centered and personalized activities, participation, and communication, "In the moment" (allowed participants to freely use their imaginations to reconnect and

			exploring: the role of the “individual”, “group”, and the role of space, place, and objects.	theatre, storytelling, creative writing, or visual arts)<br>• outcome measures (effectiveness, participation, or health and wellbeing)<br>• research designs including quantitative, qualitative, or mixed methods<br>• in English<br>• publication date between 2008 and 2019.<br><br>Exclusion:<br>• advanced dementia<br>• residential care settings, hospitals, and hospices<br>• therapy or clinical based<br>• One-to-one interventions not in a group setting.<br>• Invasive/biological testing or measurements using blood sample, x-rays, or scans.<br>• other formats of publication (book reviews, commentaries, literature reviews and meta-analyses, dissertations, or theses)		make sense of the fragments that remain as aspects in person), attention and cognitive stimulation.<br><br>The group found importance on social cohesion and togetherness and in new and existing relationships.<br><br>Space, place, and objects underlined the benefits in semi-public spaces where there are less distractions, compared to a residential home, and provided a sense of societal contribution.
Zeilig et al. (2014)	Not stated	Dementia and Alzheimer’s Disease	To strengthen the knowledge surrounding the efficacy of art-based approaches for persons with dementia and to investigate the question:<br>1. What is the value of arts and culture for people living with a dementia?	Inclusion:<br>• reference to creative activities that involved persons with dementia<br>• role of museums and/or art galleries<br>• participatory arts activities that aim to enhance well-being or quality of life<br>• participatory arts activities that focused on aesthetic appreciation<br>• activities that did not include therapies of any kind<br>• systematic data/meta-analyses about the value of the arts for older people, including one Cochrane Review on music therapy for people with dementia	63	Participatory arts contribute positively to persons with dementia, including improving communication, encouraging residual creative abilities, new learning, enhancing cognitive function, increasing confidence/self-esteem/social participation, and sense of freedom in creation.<br><br>Arts act as a way to uncover and communicate with the inner worlds of the persons with dementia.

Dance Therapy

Study	Setting	Population	Research Aims	Eligibility	Included Studies	Key Findings
Jiménez et al. (2019)	Not stated	Adults aged 60 and older with a mental health disorder	The aim of this systematic review was to identify and appraise studies on dance movement therapy interventions with adults aged over 60, who have mental health disorders.	Inclusion:<br>• people from 60 years of age with mental disorders<br>• dance movement therapy<br>• studies that reported patient relevant parameters and patient relevant data<br>• published empirical studies (quantitative and qualitative) or systematic reviews.<br>• in English, French, German, or Spanish<br><br>Exclusion:<br>• studies that evaluated dance inventions without involvement of a qualified dance movement therapist<br>• studies on expert opinions, literature reviews which were not systematically conducted<br>• grey literature	16	Majority of studies were of older people with dementia; one study focused on depression.
Karkou & Meekums (2017)	No restrictions	People with dementia	The authors aimed to assess the effects of dance movement therapy on behavioural, social, cognitive, and emotional symptoms of people with dementia in comparison to no treatment, standard care, or any other treatment. They also aimed to compare different forms of dance movement therapy.	Inclusion:<br>• randomized controlled trials<br>• people with dementia – no age or setting restrictions<br>• any language<br>• dance movement therapy delivered by a therapy practitioner with formal training<br>• included both group, individual, and family/couple dance movement therapy	0	Zero studies were included.<br><br>The authors believed that relevant trials may exist in the grey literature. Possibility exists that dance movement therapy is defined differently in each country based on history, legal frameworks, and training.
Klimova et al. (2017)	Not stated	People with dementia	The purpose of this study is to explore the efficacy of dancing on people with dementia, as well as listing the benefits and limitations of dancing therapy for this population.	Inclusion:<br>• explored sources of clinical trials, qualitative descriptive studies, review studies<br>• reports written exclusively in English<br>• focused on the issues of dancing therapy with respect to dementia<br>• years of 2010 up to 2015	6	Benefits of dancing include: improved physical state, cognitive functions, physiological health, social interaction, autonomy, quality of life, a type of non-invasive treatment, lower costs, and delay of institutional care, reduces caregivers' burden

						Limitations of dancing include: lacks randomized clinical trials, small sample sizes, short-term trials, non-pharmacological therapies are shown to be more effective for middle-aged health individuals
Mabire et al. (2019)	Not Stated	Dementia	This review aimed to a) analyze studies on dance interventions for people with dementia and b) identify practice recommendations for the development of these interventions.	Inclusion:<br>• any study design that used dance interventions for people with dementia<br>• dance interventions can be combined with other interventions if dance was the main intervention	14	Four categories of processes (physical, cognitive, psychological, and social) were seen in the studies.<br><br>None of the included studies compared the effectiveness of different styles of dance.<br><br>See table 4 in Mabire et al. (2019) for list of practice recommendations.
Ruiz-Muelle & López-Rodríguez (2019)	Not stated	Alzheimer's Disease	The purpose of this study is to review the current literature identifying clinical trials that explore the effects of dancing on psychological and physical outcomes, functionality, cognitive function, and quality of life in patients diagnosed with Alzheimer's disease. Additionally, the review wishes to assess the quality of studies that perform dance therapy.	Inclusion:<br>• pilot or clinical trials<br>• effect of dancing on people with Alzheimer's disease<br>• in English, French, Portuguese, Italian or Spanish<br>• no age, gender, or stage of illness restrictions<br>• published from July 2000	12	The type of dancing intervention impacts the person different (e.g., Waltz versus elder clown)<br><br>Frequency and duration of dancing intervention varied.<br><br>Outcomes including physical outcomes, functionality, cognitive function, psychological outcomes, quality of life, burden of care, and other outcomes (language, ethnic interaction, and ethnic identity), influenced persons with Alzheimer's disease differently.

Music Therapy

Study	Setting	Population	Research Aims	Eligibility	Included Studies	Key Findings
Aleixo et al. (2017)	Nursing or residential homes; in treatment in-day center, tertiary hospital and hospice program	Dementia, AD, VAD, mixed dementia with mild-severe severity	Assessed the efficacy of Music Therapy in the neuropsychiatric symptoms of people with dementia.	Inclusion:<br>• Alzheimer's Disease, Vascular Dementia, mixed or frontotemporal dementia, in all stages of severity.<br>• cross-sectional or longitudinal studies, randomized or nonrandomized, with or without a control group	12	Studies indicated the efficacy of music therapy on the decline of delusions, agitation, anxiety, apathy, irritability, aberrant motor activity, night-time disturbances, other neuropsychiatric symptom, behaviour, and depression. Cognitive functioning improved.<br><br>Various assessments tools were used to assess neuropsychiatric symptoms.

				The studies were categorized according to its efficacy on the neuropsychiatric symptoms.<br><br>Exclusion:<br>• with no casuistic, clinical trials about pharmacologic interventions<br>• studies about MT among people with other pathologies or children, young adults or elderly people without dementia<br>• studies developed in mental health institutions,<br>• use of music or other non-pharmacological interventions only with caregivers		Studies used active group, active individual, receptive group, and receptive individual Music Therapy interventions.
Blackburn, & Bradshaw (2014)	Residential Care	Dementia/AD	Identify if Music Therapy is beneficial for use with dementia patients.	Inclusion:<br>• randomized controlled trials<br>• participants diagnosed with dementia as defined by the Mini-Mental State Examination or equivalent diagnostic rating scale<br>• conducted in residential care<br><br>Exclusion:<br>non-English-language publications.	6	Two studies utilized passive MT techniques. Four studies used active MT interventions.<br><br>Methodological quality may be considered a weakness. However, findings suggest that Music Therapy *may* reduce depression, anxiety, agitation, and improved cognitive functioning and quality of life.
Chatterton et al. (2010)	Residential care facility and at home	Caregivers, Music Therapists, Persons with Dementia	This review sought to investigate who sings to people with dementia and with what objectives and effects to answer: which is more effective, the singer or the singing?<br><br>Sub-questions were: who sings individually with PEOPLE WITH DEMENTIAs? What were their objectives and effects?	Inclusion:<br>• one-to-one singing interactions with a people with dementia using only live singing, not recorded music<br>• in English<br>• full texts<br><br>Exclusion:<br>• review articles<br>• if singing was undertaken in a situation other than individual person-to-person<br>• articles describing an individual people with dementia singing or being sung to while part of a group were not included	16	It was noted that caregivers' primary objective using singing was to improve the quality of life. Whereas music therapists sought to address cognitive, behavioural, physiological, and social factors through one-to-one singing.<br><br>More weight fell on the singing versus the singer based on the included studies. However, singing interventions depend on both the goals of the singers and perceptions of the persons with dementia.

Clare & Camic (2019).	Residential and Community	Dementia	Research questions:<br>1. What impact does group music intervention, with active participation, have on: cognitive function, behavioural or psychological symptoms, physiological responses, quality of life and communication?<br>2. Is there a difference in impact on these outcomes according to whether live or recorded music is used?	Inclusion:<br>• group music<br>• intervention focused on music rather than related interventions such as singing on its own<br>• the intervention involved an opportunity for active participation using instruments<br>• participants were people with any type of dementia<br>• peer reviewed works in English	15	Positive impact seen on behavioural and psychological 201, quality of life, communication, and some aspects of cognitive function.<br><br>The use of recorded music resulted in more positive behavioural and psychological outcomes. Interventions using live music reported a benefit to communication and relationships.
Dowson et al. (2019)	Not Stated	Various types of Dementia with a range of severity	The aim of this paper was to provide a detailed picture of music and dementia published since 1980, including important contextual data about study design, sample size, types of dementia and where music interventions take place.	Inclusion:<br>• English, peer-reviewed between 1980-2018<br>• research with people with dementia<br>• use of live or recorded music intervention where the goal is to promote health and/or well-being<br>• music intervention paired with another intervention can be included only if musical component was distinguishable<br>• if studies used participants with and without dementia, results were included for persons with dementia<br><br>Exclusion:<br>• other types of articles were excluded if they were not clear on participants or intervention	163	Evaluated domains included music-related responses, quality of life, neuropsychiatric symptoms, cognitive function, and physiological changes (see Figure 2 in Dowson et al., 2019).<br><br>Findings show that neuropsychiatric symptoms have been prioritized in music and dementia research, however music has the potential to support and strengthen relationships, provide 'in the moment' experiences, and the meet the psychosocial needs of people with dementia.
van der Steen et al. (2018)	Nursing homes, residential homes and hospital wards	Persons with dementia included according to Diagnostic and Statistical Manual of Mental Disorders-IV,	The review aimed to assess the effects of music-based therapeutic interventions on the emotional well-being, quality of life, mood disturbance or negative affect, behavioural problems, social	Inclusion:<br>• randomized controlled trials of music-based therapeutic interventions (at least five sessions)<br>• measured outcomes of interest (van der Steen et al., 2018, pp. 7-8)<br>• control groups either received usual care or other activities with or without music	22	Providing at least five sessions of music-based therapeutic intervention probably reduces depressive symptoms and improves overall behavioural problems at the end of treatment.<br><br>Sessions may improve emotional well-being and quality of life and reduce anxiety, however, they may have little or

		International Classification of Diseases-10, or other accepted diagnostic criteria	behaviour, and cognition for persons with dementia at the end of therapy and four or more weeks after the end of treatment	• diverse settings, all severities of dementia, and no age restrictions<br>• active or receptive music interventions delivered in groups or to individuals<br>• music intervention met *at least* two of the following criteria/indicators: 1. therapeutic objective which may include communication, relationships, learning, expression, mobilization, and other relevant therapeutic objectives; 2. music matches individual preferences; 3. active participation of the people with dementia using musical instruments or singing; 4. participants had a clinical indication for the intervention or were referred for the intervention by a clinician.<br>• music should be a main element of the intervention<br>• control groups could receive activities in which music was used, but they could not receive any music-based therapeutic intervention<br>• all assessments tools accepted<br>• outcomes were sought a minimum of four weeks after the treatment to assess long-term effects		no effect on agitation, aggression, or cognition. Uncertainty on social behaviour and about long-term effects.

Psychosocial interventions

Study	Setting	Population	Research Aims	Eligibility	Included Studies	Key Findings
Lawrence et al. (2012)	Nursing Home	Dementia	Through a qualitative synthesis, understanding the successful in implementing psychosocial interventions in care homes.<br><br>See page 345 for a list of psychosocial interventions (Lawrence et al. 2012)	Inclusion:<br>• English-language articles that reported qualitative research<br>• articles looking at the use and effectiveness of psychosocial interventions from perspectives with persons with dementia, relatives, or care staff<br>• no restrictions placed on type of dementia<br>• participants residing in a residential facility	39	Benefits associated with a psychosocial intervention: enabled people with dementia to connect with others; enabled the feeling of making a meaningful contribution; provided an opportunity to reminisce<br><br>Focus on staff: supervision provided as a way to reflect on caregiving style and aided for some to look beyond the illness.

<u>Sensory interventions</u>

Study	Setting	Population	Research Aims	Eligibility	Included Studies	Key Findings
Smith & D'Amico (2020)	Residential Facilities	Dementia and Alzheimer's Disease	The purpose of this review was to explore the evidence of sensory-based occupational therapy interventions persons with dementia and Alzheimer's Disease.	Inclusion:<br>• published between 2002 and September 2017<br>• full-text English and peer-reviewed<br>• sensory-based intervention, within the occupational therapy scope of practice<br><br>Exclusion:<br>• studies based in the community or home, extenuating author biases (monetary benefit), and study participants with compounding diagnoses such as schizophrenia	47	Sensory- based therapy focused on vision, auditory, olfactory, gustatory, vestibular, and tactile.<br><br><u>Sensory Stimulation:</u><br>Light (vision)<br>Aromatherapy (olfactory)<br>Massage (tactile)<br>Snoezelen (multi-sensory stimulation)<br><br><u>Environment-based intervention:</u><br>Art (visual environment-based intervention)<br>Music (auditory)<br>Visual + Auditory<br><br><u>Occupation-based intervention:</u><br>dance and yoga (proprioceptive & vestibular)<br>gardening (occupation based)<br>mealtime (gustatory and occupation)<br>animal assisted therapy (tactile)<br>Montessori intervention (multi-sensory and occupation based)
