# Supplementary material for: A Brief Report on Reviews of Existing Creative Art–Based Interventions in Dementia Care From 2010–2020
Source: Front Aging. 2022 Apr 28;3:865533. doi: 10.3389/fragi.2022.865533 (PMC9261444; doi:10.3389/fragi.2022.865533)
Supplement: Supplementary file 2 [file Table2.pdf]

Supplementary File SB

Assessment tools found by the reviews studies (on included studies).

Study	Assessment Tools	
Art Therapy		
Beard (2011)	Mini-Mental State Examination (MMSE)<br>Cohen- Mansfield Agitation Inventory (CMAI)<br>Clinical Dementia Rating (CDR)<br>Geriatric Depression Score (GDS)<br>Dementia Care Mapping (DCM)<br>Musical Therapy Assessment Tool<br>Residual Music Skills Test<br>Greater Cincinnati Chapter Well-Being Observation Tool	
Brown Wilson et al. (2019)	Rating Anxiety in Dementia (RAID)<br>Neuropsychiatric Inventory (NPI) or Nursing Home (NPI-NH)<br>Behavioral Pathology in Alzheimer's Disease (BEHAVE-AD)<br>The Dutch version of the subscale Anxiety from the Symptom Check List (SCL-90)<br>Hamilton Scale<br>Philadelphia Geriatric Centre Affect Rating Scale<br>Campbell scale	
Cavalcanti Barroso et al. (2020)	Greater Cincinnati Chapter Well-Being Observation Tool	
Chancellor et al. (2014)	Neuropsychiatric Inventory (NPI), Cohen-Mansfield agitation scale, Quality of Life Scale in Alzheimer's Disease are suggested.	
Cowl & Gaugler (2014)	N/A	
Deshmukh et al. (2018)	Mini-Mental State Examination (MMSE)<br>Wechsler Memory Scale-Revised (WMS-R)<br>Sub-scale evaluating logical memory (WMS-log).<br>Geriatric Depression Scale (GDS)<br>Apathy Scale (Japanese version)<br>Mental Component Summary (MCS-8)<br>Dementia Behaviour Disturbance Scale (DBD)<br>Tests of Everyday Attention (TEA)<br>Benton Fluency Task (BFT)	Barthel Index (BI)<br>Zarit Caregiver Burden Interview (CBI)<br>Cornell Scale for Depression in Dementia (CSDD)<br>Multi Observational Scale for the Elderly (MOSES)<br>Rivermead Behavioural Memory Test (RBMT)<br>sub-test to assess short-term memory (RBM-STM)
Salisbury et al. (2011)	Greater Cincinnati Chapter Well-Being Observation Tool<br>Mini-Mental State Examination (MMSE)<br>Emotion Spectrum Analysis Method (ESAM)	
Ward et al. (2020)	N/A	
Zeilig et al. (2014)	Dementia Quality of Life and Geriatric Depression Scales<br>Addenbrooke Cognitive Examination<br>Geriatric Depression Scale<br>Dementia Quality of Life (Dem-QOL-proxy)<br>Neuropsychiatric Inventory<br>Bristol Activities of Daily Living Scale	

Dance Therapy		
Jiménez et al. (2019)	N/A	
Karkou & Meekums (2017)	N/A	
Klimova et al. (2017)	N/A	
Mabire et al. (2019)	Mental State Examination (MMSE)<br>Clinical Dementia Rating (CDR)<br>Tinetti Balance Tool<br>Clack Drawing Test	
Ruiz-Muelle & López-Rodríguez (2019)	Berg Balance Scale (BBS)<br>Timed Up and Go Test (TUG)<br>Agility/dynamic Balance test (AGIBAL)<br>Mini-Mental State Examination (MMSE)<br>Clock Drawing Test<br>Cookie Theft picture from Boston Diagnostic Aphasia Test<br>Nurses' Observation Scale for Geriatric Patients (NOS-GER)<br>Montreal Cognitive Assessment Test (MOCA)<br>Geriatric Depression Scale (GDS)<br>Beck Depression Inventory (BDI)<br>Hamilton Scale<br>Perceived Stress Scale (PSS)<br>Beck Anxiety Inventory<br>Functional Rating Scale for Dementia (FRSSD)<br>Functional and Cognitive Assessment Test (FUCAS)<br>Trail Making Test part-B (TRAIL-B)<br>Rey Auditory Verbal Learning Test (RAVLT)<br>Test of Everyday Attention (TEA)<br>Acculturation Rating Scale for Mexican Americans-II (ARSMA-II)<br>Trail Making Test (TMT)<br>Stroop Neuropsychological Screening Test (SNST)<br>Word fluency test<br>Symbol Digit Modalities Test<br>Digit Span test/Digit Ordering test<br>Logical Memory I and II<br>Modified Mini-Mental State Examination (3MS)	Quality of Life Scale in Alzheimer's Disease (QoL-AD)<br>The General Health Questionnaire (GHQ-12)<br>Neuropsychiatric Inventory (NPI)<br>Hospital Anxiety and Depression Scale (HAD)<br>Barthel Index (BI)<br>Scale ADL-Katz<br>Senior Fitness Test (SFT)<br>Neuropsychiatric Inventory- Nursing Home Version (NPI-NH)<br>Dementia Care Mapping (DCM)<br>Modified Nursing Care Assessment Scale (M-NCAS)<br>Cohen-Mansfield Agitation Inventory (CMAI)<br>Alzheimer's disease Cooperative Study-Activities of Daily Living (ADCS-ADL)<br>Activities of Daily Living Katz Scale (ADL-Katz Scale)<br>Social Competence subscale<br>Rey Osterrieth Complex Figure Test (ROCFT)<br>Color Task (Stroop C), Color-Word task (Stroop C-W)<br>Rivermead Behavioral Memory Test (RBMT)<br>Verbal Fluency F-A-S test (FAS)<br>Short Physical Performance Battery (SPPB)<br>Senior Fitness Test (SFT)<br>Alzheimer's Disease Assessment Scale-Cognitive Subscale (ADAS-cog).
Music Therapy		
Aleixo et al. (2017)	Music Therapy Check List–Dementia (MTCL-D)<br>Music Therapy Coding Scheme (MTCS)<br>Interest in Music Evaluation Form<br>Clinical Dementia Rating (CDR)<br>Global Deterioration Scale (GDS)<br>Mini-Mental State Examination (MMSE)<br>Mental Status Questionnaire (MSQ)<br>Wechsler Adult Intelligence Scale (WAIS)<br>Neuropsychiatric Inventory (NPI)<br>Behavioral Pathology in Alzheimer's Disease (BEHAVE-AD)	Cohen-Mansfield Agitation Inventory (CMAI)<br>Revised Memory and Behavioral Problems Checklist (RMBPC)<br>Apparent Emotion Scale (AES)<br>Cornell Scale for Depression in Dementia (CSDD)<br>Chinese Version of Cornell Scale for Depression in Dementia (C-CSDD)<br>Hamilton Anxiety scale (HAM-A)<br>Barthel Index (BI)<br>Cornell-Brown Scale for Quality of Life in Dementia (CBS-QoL)

		Alzheimer’s Disease-Related Quality of Life (ADRQL)
Blackburn, & Bradshaw (2014)	Mini-Mental State Examination (MMSE)<br>Rating of Anxiety in Dementia (RAID)<br>Cohen-Mansfield Agitation Inventory (CMAI)<br>Neuropsychiatric Inventory (NPI)<br>Cornell Scale for Depression in Dementia (CSDD)<br>Agitation Behaviour Mapping Instrument<br>Dementia Quality of Life Geriatric Depression Scale<br>Chinese-Mini-Mental State Exam<br>Chinese Cohen-Mansfield Agitation Inventory	
Chatterton et al. (2010)	Mini Mental State Examination (MMSE)<br>Global Deterioration Scale (GDS)	
Clare & Camic (2019)	Mini Mental State Examination (MMSE)<br>Global Deterioration Scale (GDS)<br>Neuropsychiatric Inventory (NPI)<br>Cohen-Mansfield Agitation Inventory (CMAI)<br>Rating of Anxiety in Dementia (RAID)<br>Cornell-Brown Scale for Quality of Life in Dementia (CBS-QoL)<br>GENCAT for Quality of Life<br>Brotons&Brotons and Picket-Cooper<br>Daily living and multidimensional observation scale<br>Multidimensional Observation Scale for Elderly Subjects (MOSES)	Behavior Pathology in Alzheimer’s Disease Rating Scale (BEHAVE-AD)<br>Intelligence test – Revised Hasegawa Dementia Scale (HDS-R)<br>Geriatric Quality of Life (GQoL)<br>Cornell Scale for Depression<br>Hospital Anxiety and Depression Scale (HADS)<br>Barthel Index (BI)<br>Visual Analog Mood Scale (VAMS)<br>Cornell Scale for Depression in Dementia (CSDD)
Dowson et al. (2019)	Cohen-Mansfield Agitation Inventory (CMAI)<br>Mini-Mental State Examination (MMSE)<br>NeuroPsychiatric Inventory (NPI)<br>Cornell Scale for Depression in Dementia (CSDD)<br>Dementia Care Mapping (CDM)<br>Rating Anxiety in Dementia Geriatric Depression Scale<br>Barthel Index (BI)<br>Behavioral Pathology in Alzheimer’s Disease Rating Scale<br>Observed Emotion Rating Scale<br>Quality of Life - Alzheimer's Disease Test	Bedford Alzheimer Nursing Severity Scale<br>Cornell-Brown Scale for Quality of Life in Dementia<br>Disruptive Behaviour Rating Scale<br>Lawton's Modified Behaviour Stream<br>Montgomery-Åsberg Depression Rating Scale<br>Music in Dementia Assessment Scale<br>Menorah park engagement scale<br>Paper Folding and Cutting<br>State-Trait Anxiety Inventory<br>Test épisodique de mémoire du passé
van der Steen et al. (2018)	Cohen-Mansfield Agitation Inventory (CMAI)<br>Mini-Mental State Examination (MMSE)<br>Neuropsychiatric Inventory (NPI)<br>Dementia Quality of Life (DQOL)<br>German translation of the Dementia Quality of Life Instrument (DEMQOL)<br>Danish translation of the Alzheimer's Disease-Related Quality of Life (ADRQL)<br>Cornell- Brown Scale for Quality of Life in Dementia (CBS-QoL)<br>Dementia Care Mapping (DCM)<br>Quality of Life-Alzheimer's Disease (QOL-AD)	Geriatric Depression Scale (GDS)<br>Cornell Scale for Depression in Dementia<br>Behavioural Pathology in Alzheimer's Disease (BEHAVE-AD)<br>Rating Anxiety in Dementia Scale (RAID)<br>Hamilton Anxiety Scale<br>State- Trait Anxiety Inventory for adults (STAI-A)<br>Severe Impairment Battery (SIB)<br>Prose Memory tests, the FAS-Test (Controlled-Oral-Word-Association Test)<br>Alzheimer's Disease Assessment Scale Cognitive subscale (ADAS-cog)
Psychosocial interventions		
Lawrence et al. (2012)	N/A	

Sensory interventions

Smith & D'Amico (2020)	N/A
