# Supplementary material for: A Brief Report on Reviews of Existing Creative Art–Based Interventions in Dementia Care From 2010–2020
Source: Front Aging. 2022 Apr 28;3:865533. doi: 10.3389/fragi.2022.865533 (PMC9261444; doi:10.3389/fragi.2022.865533)
Supplement: Supplementary file 3 [file Table3.pdf]

Supplementary File C

Suggested Next Steps for future review works. These next steps may be included in search strategies and in the eligibility criteria.

Study	Next Step(s)
Art Therapy	
Beard (2011)	Consider:<br>• exploring creatives arts from the years 2010-2020<br>• including key words such as dementia and Alzheimer's Disease in the search strategy/inclusion criteria<br>• including studies that are in addition to clinical approaches<br>• expanding the search strategy to include different study designs
Brown Wilson et al. (2019)	Consider:<br>• exploring not only randomized controlled trials but other study designs as well<br>• excluding studies that are of low quality after the quality assessment stage of the review (e.g., in the ROB-2, excluding studies with a high risk based on the guidance provided by Higgins et al. (2019))<br>• investigating research that is qualitative (e.g., inclusive of the lived experiences of the target population, in addition to symptomatic change)<br>• investigating research that follows a mixed-method methodology as it provides an understanding to research problems from both quantitative and qualitative methodologies and enriches the understanding of the research problem and questions (Molina-Azorin, 2016)
Cavalcanti Barroso et al. (2020)	Consider:<br>• identifying quality assessment tools that are valid for psychosocial interventions (e.g., Cavalcanti Barroso et al. (2020) excluded items on the Downs and Black quality assessment tool as there were not applicable to psychosocial interventions (p. 23))<br>• identifying a comprehensive data synthesis strategy (e.g., narrative versus meta-analysis); ensuring that the synthesis strategy recognizes the differences between the included studies' elements of design and outcomes of the intervention
Chancellor et al. (2014)	Consider:<br>• updating from 2013<br>• evaluating the proposed three-component framework presented in this review (see pp. 4-5 in Chancellor et al. (2014))
Cowl and Gaugler (2014)	Consider:<br>• looking at other benefits of the creative arts, in addition to improving symptoms of dementia; this may enhance the review by providing a more in-depth conclusion on understanding these interventions and their usage in dementia care
Deshmukh et al. (2018)	Consider:<br>• exploring not only randomized controlled trials but other study designs as well<br>• expanding searches to more than one database (e.g., Deshmukh et al. (2018) only searched within ALOIS but may have benefitted from searching in Web of Science and/or PsycINFO, especially for dementia-related studies)
Salisbury et al. (2011)	Consider:<br>• investigating research that follows a mixed-method methodology<br>• expanding search parameters to include the years after 2010

	• the suggestions that Salisbury et al. (2011) made on conducting research on effectiveness and cost analysis but also valuing the worth of the persons with dementia (benefit versus recovery or treatment)
Ward et al. (2020)	Consider:<br>• continuing to have no study design restrictions<br>• the health and well-being of the persons with dementia (similar to Salisbury et al. 2011: benefit versus recovery or treatment)<br>• the way creative arts are conducted for all stages of dementia in addition to design and facilitation at a practical level (see Ward et al., 2020, p. 17)
Zeilig et al. (2014)	Consider:<br>• expanding on participatory arts to include creative arts-based interventions including art, music, and drama which were missing in Zeilig et al. (2014, p. 10)
Dance Therapy	
Jiménez et al. (2019)	Consider:<br>• including persons who facilitate dance with all or no qualifications (exclusion criteria of Jiménez et al. (2019))
Karkou and Meekums (2017)	Consider:<br>• exploring not only randomized controlled trials but other study designs as well (Karkou and Meekums (2017) yielded zero studies with the randomized controlled trials restriction)<br>• including all persons who facilitate or delivery dance movement therapy (i.e., may provide as a comparison between effectiveness in the delivery)
Klimova et al. (2017)	Consider:<br>• expanding the search strategy to include different study designs<br>• expanding for the years 2016 and onwards
Mabire et al. (2019)	Consider:<br>• investigating what symptoms or outside factors can impact the four processes mentioned by Mabire et al. (2019)<br>• understanding the effectiveness of different styles of dance for persons with dementia (similar to what Karkou and Meekums (2017) attempted to accomplish)
Ruiz-Muelle and López-Rodríguez (2019)	Consider:<br>• expanding the search strategy to include different study designs<br>• including key words such as dementia and Alzheimer's Disease in the search strategy/inclusion criteria<br>• continuing to not set age, gender, or stage of illness restrictions; may help gather enough information to compare the benefits of dance amongst age groups, gender, and stages of dementia
Music Therapy	
Aleixo et al. (2017)	Consider:<br>• expanding the eligibility criteria to look at other symptoms of dementia and to see if they may or may not improve with music therapy<br>• continuing to be inclusive to all forms and stages of dementia and to the different study designs (e.g., Aleixo et al. (2017) included cross-sectional or longitudinal studies, randomized or nonrandomized, with or without a control group)

Blackburn and Bradshaw (2014)	Consider:<br>• exploring not only randomized controlled trials but other study designs as well (Blackburn and Bradshaw (2014) yielded six studies with the randomized controlled trials restriction)
Chatterton et al. (2010)	Consider:<br>• including studies that looked at individual person-to-person singing and in-group singing; may provide data that will allow reviewers to compare the effectivity between the type of interactions and the benefit that each environment may provide to the person with dementia (Chatterton et al. (2010) excluded studies that did not include individual person-to-person singing)<br>• investigating the effects of singing provided to persons with dementia by other persons, not only caregivers and music therapists; including, music students, family*, or staff<br><br>**note: not all family members or staff are usual caregivers*
Clare and Camic (2019)	Consider:<br>• investigating not only behavioural and psychological outcomes, but other outcomes that may impact the health and well-being of the person with dementia
Dowson et al. (2019)	Consider:<br>• including outcome measures on well-being/quality of life factors that are inclusive of all the symptoms of dementia and their impact on the person with dementia (in the search strategy); including a person-centred lens when addressing these outcomes<br>• listening to people with dementia and their carers when designing studies (as suggested by Dowson et al. (2019, p. 30)); in other words, engagement with the necessary stakeholders to create studies that will be beneficial to them as well
van der Steen et al. (2018)	Consider:<br>• changing the minimum number of sessions of music-based therapeutic interventions (van der Steen et al. (2018) stated at least five minimum sessions in their eligibility criteria)<br>• what time is appropriate for post-intervention follow-up (van der Steen et al. (2018) looked for a minimum of four weeks, which may have excluded studies)
Psychosocial interventions	
Lawrence et al. (2012)	Consider:<br>• continuing to pull data that is looking at the effectiveness of the creative arts and perspectives of people with dementia, as well their carers (i.e., including different viewpoints)<br>• including mixed-methods and quantitative research with the same eligibility found in this study (Lawrence et al. (2012) included only qualitative research)<br>• continue involving participants with any type of dementia<br>• involving persons with dementia living in multiple living environments (Lawrence et al. (2012) looked at participants in residential facilities only)
Sensory interventions	
Smith and D'Amico (2020)	Smith and D'Amico (2020) view their evidence of sensory-based occupational therapy interventions.<br><br>Consider:<br>• envisioning evidence from another lens, in addition to occupational therapy (perhaps another clinical approach (e.g., psychotherapy), academia, or government reporting)
